# The Relationship between Plasma Pentraxin 3 and Serum Amyloid P Levels and Disease Activity in Ankylosing Spondylitis

**DOI:** 10.1155/2022/7243399

**Published:** 2022-03-24

**Authors:** Ali Erhan Özdemirel, Serdar Can Güven, İsmihan Sunar, Zühre Sari Sürmeli, Alper Doğanci, Hüseyin Tutkak, Ayşe Peyman Yalçin Sayin, Şebnem Ataman

**Affiliations:** ^1^Gaziler Physical Therapy and Rehabilitation Training and Research Hospital, Clinic of Rheumatology, Ankara, Turkey; ^2^Ankara City Hospital, Clinic of Rheumatology, Ankara, Turkey; ^3^Aydın State Hospital, Clinic of Rheumatology, Aydın, Turkey; ^4^Memorial Şişli Hospital, Clinic of Rheumatology, İstanbul, Turkey; ^5^ErzurumRegional Training and Research Hospital, Clinic of Physical Therapy and Rehabilitation, Erzurum, Turkey; ^6^Ankara University, Faculty of Medicine, Department of Immunology and Allergy, Ankara, Turkey; ^7^Ankara University, Faculty of Medicine, Department of Physical and Rehabilitation Medicine, Ankara, Turkey; ^8^Ankara University, Faculty of Medicine, Department of Physical and Rehabilitation Medicine, Division of Rheumatology, Ankara, Turkey

## Abstract

**Background:**

In clinical practice, it is hard to judge the level of disease activity in some patients with ankylosing spondylitis (AS) who have low traditional acute phase reactant values such as erythrocyte sedimentation rate (ESR) and C-reactive protein (CRP) but have considerable pain and inflammation. The aim of this study is to investigate plasma pentraxin 3 (PTX3) and serum amyloid P (SAP) levels in patients with AS who had normal ESR and CRP but high disease activity.

**Methods:**

100 AS patients and 100 gender- and age-matched controls were included. Epidemiological, clinical, and treatment data and plasma levels of CRP, ESR, PTX3, and SAP were evaluated. The Bath Ankylosing Spondylitis Disease Activity Index (BASDAI) and the Ankylosing Spondylitis Disease Activity Score (ASDAS)-CRP were used for evaluating disease activity. Plasma levels of PTX3 and SAP were compared between AS patients and controls and also among AS patients with active and inactive disease.

**Results:**

AS patients had significantly higher plasma levels of PTX3 and SAP than controls. There were not any significant correlations between PTX3 and SAP with BASDAI, ASDAS-CRP, and ESR. There was a positive correlation between PTX3 and CRP. No significant difference in plasma levels of PTX3 and SAP was observed between patients with active disease and inactive disease, both with normal ESR and CRP levels. Disease duration and treatment did not influence plasma PTX3 levels.

**Conclusions:**

In patients with AS, plasma levels of PTX3 and SAP were found to be elevated when compared to healthy controls. No association was observed between these biomarkers and disease activity.

## 1. Introduction

Ankylosing spondylitis (AS) is a chronic inflammatory disease involving mainly sacroiliac joints, the spine, and entheses in which bone erosion as well as new bone formations and ankylosis occur as a result of chronic inflammation [1]. Assessment of disease activity in AS is quite complicated since a certain relationship between acute phase reactants, clinical and imaging findings, and the progression of disease is lacking [2, 3]. The *Bath Ankylosing Spondylitis Disease Activity Index* (BASDAI) is a subjective assessment scale for the determination of disease activity in AS [4]. *Ankylosing Spondylitis Disease Activity Score* (ASDAS) is a disease activity scale that is relatively more objective than BASDAI as it involves either C-reactive protein (CRP) or erythrocyte sedimentation rate (ESR), and is widely used in estimating disease activity for AS [2]. ESR and CRP are acute phase reactants useful in evaluating disease activity in AS [2–4]. However, among AS patients with active disease, only 50–70% have increased levels of CRP and ESR, therefore their levels may not always correlate with disease activity [2–4].

Pentraxins are a superfamily of multifunctional proteins involved in chronic inflammation which also act as acute phase reactants. Serum amyloid P (SAP), CRP, and pentraxin 3 (PTX3) are examples for the well-known pentraxins. In case of inflammation and trauma, PTX3 is produced and released by the immune cells such as dendritic cells, mononuclear phagocytes, endothelial cells, and fibroblasts [5, 6]. SAP, a short pentraxin of 25 kDa, structurally resembles CRP, and its secretion is stimulated by proinflammatory cytokines. Nuclear autoantigens are opsonized by SAP which also interacts with inflammatory cells and the complement system [7]. SAP and PTX3 are reported to be elevated in various inflammatory rheumatic diseases [8, 9].

In clinical practice, it is challenging to assess disease activity in AS patients with low levels of traditional acute phase reactants, such as ESR and CRP, but who have considerable pain and clinically active disease. Considering that SAP and PTX3 are members of a common superfamily with CRP and the role of CRP in evaluating disease activity in AS, our aim in this study was to investigate whether PTX3 and SAP might be useful as markers for disease activity in AS patients who have normal ESR and CRP levels despite having clinical findings suggestive of high disease activity.

## 2. Materials and Methods

A total of 100 consecutive AS patients meeting the criteria of Modified New York who attended Ankara University Medical School, Physical Medicine and Rehabilitation Department, Rheumatology Division, were included in this study between January, 2014 and January, 2015. A control group was formed by 100 healthy volunteers that who matched with the patients in terms of age and gender.

In accordance with the Helsinki Declaration, local ethics committee approval and written informed consent from participants were obtained before study procedures. A cross-sectional study was conducted. Demographic and clinical data were recorded during routine follow-ups. Patients were subgrouped, as those under antitumor necrosis factor-alpha (anti-TNF-*α*) treatment and those under nonsteroidal anti-inflammatory drugs (NSAIDs). Patients who did not use more than one NSAID per week with anti-TNF-*α* were included in the first group. The exclusion criteria were set as follows: age under 18 and over 55, presence of pregnancy, malignancy, acute infection, secondary amyloidosis, severe hepatic, renal, or cardiac disease, concomitant with any other rheumatic disease, peripheral joint involvement at the time of enrollment, history of conventional DMARD use in the last 6 months, corticosteroid use in the last 3 months, and NSAID use more for than a week despite anti-TNF treatment.

BASDAI and ASDAS-CRP were used for the assessment of disease activity. Patients with a BASDAI score of ≥4 were considered as having active disease, while those with a BASDAI score <4 were considered to be in remission. Patients with an ASDAS-CRP score of <1.3 were accepted to be in remission, ≥1.3 to <2.1 to have mild disease activity, ≥2.1 to <3.5 to have moderate disease activity, and ≥3.5 to have severe disease activity.

ESR and CRP values were examined routinely as inflammatory markers in follow-ups. Venous blood samples were also obtained following a minimum of 8 hours of fasting to determine plasma SAP and PTX3 levels. The samples for SAP and PTX3 analysis were collected in sterile containers and centrifuged within a maximum of 120 minutes at 4000 rpm for 10 minutes. All samples were stored at −80°C until the analysis. Plasma SAP and PTX3 measurements were performed with an enzyme-linked immuno sorbent assay (ELISA) commercial kit in accordance with the manufacturer's guidelines (Boster Immunoleader, Fremont, CA, USA). The sensitivity of the PTX3 kit and SAP kit was <10 pg/ml.

Statistical Package for Social Sciences version 21.0 (SPSS Inc., Chicago, IL, USA) software was used for statistical analyses. The minimum number of patients per group was calculated as 100 when the alpha error was 0.05, the beta error was 0.20, and the effect size was 0.40. The variables were investigated using visual (histograms and probability plots) and analytical methods (Kolmogorov–Smirnov test and Shapiro–Wilk test) to determine normal distributions. Continuous variables were presented either using means ± standard deviations (SD) or medians (minimum and maximum) according to normality. The categorical data were presented with numbers and percentages. The Mann–Whitney-U test was used to compare two independent groups. The Kruskal–Wallis test was performed to compare 3 or more independent groups, and the Bonferroni-corrected Mann–Whitney-U test was used to evaluate the parameters with significant differences. A Chi-square test was used to compare the categorical variables. The correlation of markers with disease activity parameters was evaluated by Spearman's Rho. *p* values <0.05 were considered statistically significant in all analyses.

## 3. Results

A total of 200 subjects (100 AS patients and 100 healthy volunteers) were included in the study. The AS group comprised 61 males and 39 females with a median (min-max) age of 40.5 (18–54) years, and the control group comprised 56 males and 44 females with a median (min-max) age of 38 (18–51) years. The mean ± SD disease duration in patients with AS was 8.33 ± 4.19 years, the median (min-max) ESR was 12.5 (1–76) mm/hour and the median (min-max) CRP was 2.9 (1–61.5) mg/dL in the AS group.

When the AS and control groups were compared in terms of PTX3 and SAP levels, significantly higher values, especially in PTX3, were observed in the AS group compared to the control group (*p* < 0.001 and *p*=0.015, respectively) (Table 1). There was no statistically significant correlation between PTX3 and SAP levels and disease activity scores (BASDAI and ASDAS-CRP) and acute phase reactants (ESR and CRP), except for a positive weak correlation between PTX3 and CRP (*r* = 0.220, *p*=0.028) (Table 2).

AS patients were subgrouped according to the BASDAI and ASDAS-CRP scores, thereafter. 30 patients with a BASDAI score of ≥4 constituted the active disease group, and 70 patients with a BASDAI score of <4 constituted the inactive disease group. There were no significant differences in PTX3 and SAP values between the two groups (*p* > 0.05). When patients were grouped as being in remission (*n* = 22), having low disease activity (*n* = 34), high disease activity (*n* = 40), or very high disease activity (*n* = 4) according to the ASDAS-CRP scores, no significant difference was observed in PTX3 and SAP levels between groups (*p*=0.920 and *p*=0.673, respectively) (Table 3).

Both ESR and CRP values were within the normal range in 53 patients with AS. When these patients were classified as inactive (<4) and active (≥4) according to the BASDAI score, no significant difference in any of the markers was detected between the groups (*p*=0.576 and *p*=0.911, respectively) (Table 4).

When patients with AS were examined according to the treatment agents, 71 were found to be receiving anti-TNF-*α* treatment (adalimumab *n* = 24, etanercept *n* = 22, infliximab *n* = 15, and golimumab = 10), and the mean ± SD treatment duration was 5.8 ± 3.21 years. A total of 29 patients with AS were under NSAIDs, and the mean ± SD duration of treatment was 6.3 ± 3.39 years. There was no difference in PTX3 and SAP levels between groups receiving anti-TNF-*α* and NSAID therapy (*p*=0.623 and *p*=0.172, respectively) (Table 5). Additionally, no significant correlations were found between PTX3 and SAP levels and the duration of treatment (*r* = 0.112, *p*=0.473 and *r* = 0.087, *p*=0.719, respectively).

## 4. Discussion

We found PTX3 and SAP levels, especially PTX3, significantly higher in patients with AS compared to the control group. On the other hand, there were no correlations between PTX3 and SAP levels and disease activity assessed by ASDAS-CRP and BASDAI, whereas a significant weak positive correlation between PTX3 and CRP was observed. We also observed that PTX3 and SAP levels were not affected by treatment modalities.

ESR and CRP are extensively used in the diagnosis and follow-up of rheumatic diseases, infections, and malignities. Elevations in acute phase reactants may indicate active disease also in AS. In addition, there are other acute phase reactants such as alpha-1-antitrypsin, immunoglobulin A, G, and M, interferon-gamma, complement 3, complement 4, and interleukin (IL)-4 which are useful for assessment of disease activity in AS [5, 10, 11]. To our best knowledge, there is only one study in the literature that examined PTX3 levels and disease activity in patients with AS [4]. However, the number of patients was relatively low, and only BASDAI was used for the assessment of disease activity [4]. Similar to our findings, the authors declared high PTX3 levels in patients with AS, but it was not associated with disease activity [4]. In the current literature, there is no study examining the relationship between SAP and AS in terms of disease activity.

In our study, plasma PTX3 levels were significantly higher in patients with AS when compared to the control group, but there was no relationship between PTX3 and disease activity. Similar results were valid in terms of SAP levels. These results may suggest that these markers are associated with chronic inflammation rather than disease activity. Accordingly, when the correlations between PTX3, SAP and ESR, and CRP were analyzed, the latter two of which elevate rather faster in the case of inflammation, only a weak correlation was found between PTX3 and CRP. Furthermore, when patients with AS were subdivided according to the level of disease activity according to BASDAI and ASDAS-CRP, there were no differences in PTX3 and SAP levels between the groups. In addition to this, when patients with normal ESR and CRP levels were subdivided as active and inactive according to BASDAI scores, no differences were found in SAP and PTX3 levels between groups. Finally, the SAP and PTX3 values in our patients were not affected by different treatment modalities (NSAID or anti-TNF-*α*) which suggested that these markers might be irrelevant for assessment of active inflammation. On the other hand, in our study, the diagnosis of patients was based on the modified New York criteria which implied a relatively long disease duration, avoiding the assumptions about the utility of these markers as signs of disease activity in the early disease period. Since SAP and PTX3 levels seem not to be affected by disease activity, these markers may be useful to differentiate AS patients from noninflammatory low back pain patients, even if they are in remission.

One of the main objectives of our study was to determine whether PTX3 and SAP levels could be indicators for high disease activity despite normal ESR and CRP values. ESR and CRP are the most frequently used markers in the evaluation of disease activity and treatment response [2]. Nevertheless, just 60% of patients with active AS have elevated levels of acute phase reactants [12]. Acute phase reactants and BASDAI scores were detected to be higher in AS patients with peripheral joint involvement, but in patients with pure axial involvement, acute phase reactants were measured to be lower [2, 13, 14]. Considering this disparity, we included AS patients with pure axial involvement and no peripheral involvement in our study. When we examined AS patients with normal ESR and CRP levels, we did not observe a relationship between SAP and PTX3 levels and disease activity.

Several studies investigated the relation between PTX3 and disease activity in autoimmune and autoinflammatory conditions. Deniz et al. [4] demonstrated increased PTX3 levels in AS patients without any correlation with disease activity. Boutet et al. [15] reported increased PTX3 levels in rheumatoid arthritis patients in comparison to healthy subjects, but again, no relation with active disease was reported. Likewise, Nisihara et al. [16] did not observe any correlation between PTX levels and disease activity in spondyloarthritis patients. On the other hand, Bevelacqua et al. [17] demonstrated a relation between both plasma and intracellular levels of PTX and the severity of skin lesions in psoriasis patients. In our study, we observed increased levels of both PTX3 and SAP in AS patients, yet we could not demonstrate a relation with disease activity. These results, in accordance with the results of Deniz et al. [4], Boutet et al. [15], and Nisihara et al. [16], may imply that these biomarkers may have diagnostic value in spondyloarthritis, regardless of disease activity.

The aforementioned findings indicate an association between PTX3, SAP and chronic inflammation rather than acute inflammation; however, local inflammation may also be contributory to AS pathogenesis via both inflammation itself and consequent ossification [18, 19]. Infections, microlesions, and biomechanical stress on the entheses may also contribute to acute inflammation. In some instances, homeostasis is rebuilt and these acute events can diminish, but in particular cases, acute processes may evolve into chronic processes leading to ongoing inflammation and ossification. Genetic factors might have a role in the chronic inflammation and structural changes in joints and surrounding tissues in AS patients. Accordingly, interleukin 23 receptor gene polymorphisms (rs7517847 and rs2201841) were found to be significantly associated with AS in a meta-analysis conducted by Xu et al. [19]. Also, PTX3 polymorphisms rs3816527 and rs3845978 were found to be associated with AS, which indicates PTX3 may be a marker involved in pathogenesis rather than being solely an inflammatory marker in AS [5]. In order to support this hypothesis, studies comparing plasma PTX3 values with possible active-chronic lesions in spinal and sacroiliac magnetic resonance images, especially in patients with normal acute phase reactants such as ESR and CRP but with high disease activity scores, are needed.

## 5. Conclusion

We found that neither PTX3 nor SAP were associated with disease activity and acute inflammation in AS; however, PTX3 especially was associated with chronic inflammation. These findings should be supported by studies investigating the role of these markers, particularly in the early stages of the disease, and the relationship between these markers and local/entheseal inflammation needs to be confirmed by imaging methods in patients with normal acute phase reactants but with clinically high disease activity, in order to further elucidate the role of these markers in acute inflammation.

## Figures and Tables

**Table 1:** Distribution of pentraxin 3 and serum amyloid P values by groups.

	Ankylosing spondylitis group (*n* = 100)	Control group (*n* = 100)	*p*
PTX3, pg/mL median (min-max)	2858.8 (10–20000.0)	20.7 (10–7744.6)	<0.001
SAP, pg/mL, median (min-max)	62.2 (37.4–303.7)	55.7 (7.4–300.0)	0.015

PTX3: pentraxin 3; SAP: serum amyloid P.

**Table 2:** The correlation between pentraxin 3 and serum amyloid P levels and disease activity scores and acute phase reactants.

		ESR	CRP	BASDAI	ASDAS-CRP
PTX3	*r*	0.050	0.220	−0.088	0.053
	*p*	0.621	0.028	0.382	0.603

SAP	*r*	−0.023	−0.116	−0.170	−0.154
	*p*	0.818	0.249	0.092	0.125

PTX3: pentraxin 3, SAP: serum amyloid P, ESR: erythrocyte sedimentation rate, CRP: C-reactive protein, BASDAI: Bath Ankylosing Spondylitis Disease Activity Index, ASDAS: ankylosing spondylitis disease activity score, and *r*: Spearman's correlation coefficient.

**Table 3:** Distribution of pentraxin 3 and serum amyloid P according to the ankylosing spondylitis disease activity score C-reactive protein.

ASDAS-CRP	Inactive (*n* = 22)	Low (*n* = 34)	High (*n* = 40)	Very high (*n* = 4)	*p*
PTX3, pg/mL, median (min-max)	3103.5 (10–7485.2)	2713.7 (10–12882)	2844.2 (10–20000)	2828.7 (1102.5–5989.3)	0.920
SAP, pg/mL, median (min-max)	61.7 (42.5–300)	84.2 (37.4–300)	57.8 (40.7–303.7)	50.9 (45.2–54.7)	0.673

ASDAS: ankylosing spondylitis disease activity score, CRP: C-reactive protein, PTX3: pentraxin 3, and SAP: serum amyloid P.

**Table 4:** Distribution of pentraxin 3 and serum amyloid P according to bath ankylosing spondylitis disease activity index score in patients with ankylosing spondylitis, who had normal ESR and CRP levels.

	Inactive (*n* = 34)	Active (*n* = 19)	*p*
PTX3, pg/mL, median (min-max)	2713.7 (10–12882)	2408.1 (10–12966.5)	0.576
SAP, pg/mL, median (min-max)	67.5 (42.5–300)	89.3 (44.1–303.7)	0.911

PTX3: pentraxin 3 and SAP: serum amyloid P.

**Table 5:** Distribution of pentraxin 3 and serum amyloid P in patients with ankylosing spondylitis according to medical treatment.

	Anti-TNF-*α* (*n* = 71)	NSAID (*n* = 29)	*p*
PTX3, pg/mL, median (min-max)	2987.2 (10–20000.0)	2713.7 (10–12966.5)	0.623
SAP, pg/mL, median (min-max)	65.8 (37.4–300)	56.0 (43.1–303.7)	0.172

PTX3: pentraxin 3, SAP: serum amyloid P, anti-TNF-*α*: antitumor necrosis alpha, and NSAID: nonsteroidal anti-inflammatory drug.

## Data Availability

The data are available from the corresponding author upon reasonable request.

## Additional Points

(i) Some ankylosing spondylitis patients have active disease despite normal C-reactive protein and erythrocyte sedimentation rate levels. (ii) Pentraxins, such as serum amyloid P and pentraxin 3, also act as acute phase reactants. (ii) Serum amyloid P and pentraxin 3 may be useful markers for disease activity in ankylosing spondylitis patients with normal traditional acute phase reactant levels. *Findings.*(i) Serum amyloid P and pentraxin 3 levels were significantly higher in patients with ankylosing spondylitis compared to healthy controls. (ii) There was no correlation detected between serum amyloid P and pentraxin 3 levels and disease activity.
